# Investigation of the synergistic effect of metformin and FX11 on PANC-1 cell lines

**DOI:** 10.1186/s40659-025-00592-8

**Published:** 2025-03-17

**Authors:** Melike Bayindir-Bilgic, Ezgi Duman, Deniz Turgut, Ayse Naz Kadikoylu, Nur Ekimci-Gurcan, Utku Ozbey, Aysegul Kuskucu, Omer. F. Bayrak

**Affiliations:** 1 Department of Medical Genetics, School of Medicine, Yeditepe University, Istanbul, Turkey; 2 Department of Genetics and Bioengineering, Yeditepe University, Acıbadem Mah. Liseyolu sok. No:8 Kat: 3, Kadıköy/Istanbul, 34718 Turkey; 3 Department of Molecular Biology and Genetics, Faculty of Engineering and Natural Sciences, Biruni University, Istanbul, Turkey

**Keywords:** Metformin, Lactate, Pancreatic neoplasms, Warburg Effect

## Abstract

**Background:**

Pancreatic cancer is among the most aggressive and malignant tumors and is a leading cause of cancer-related mortality. It is characterized by its metabolic Warburg effect and glucose dependence. Aerobic glycolysis is a key feature of metabolic reprogramming in cancer cells. This study investigates the combined effect of metformin and FX11, hypothesizing that disrupting cancer cell energetics through complementary mechanisms may result in a synergistic therapeutic effect. The combination of metformin and FX11 affects the axis that regulates vital functions in cancer cells; thus, the uncontrolled growth of tumor cells, especially those that use a lactose-dependent energy pathway, can be controlled. Several in vitro experiments were conducted to evaluate this hypothesis. PANC-1 cell proliferation was assessed using an MTS assay, lactate levels were measured via an LDH assay, and apoptosis was determined using a flow cytometry-based PE-annexin V assay. The downstream effects of metformin and FX11 treatment were evaluated via western blot analysis.

**Results:**

The findings of this study revealed that metformin and FX11 significantly decreased the viability of PANC-1 cells when used in combination, and this effect was achieved by significantly affecting the energy mechanism of the cells through the AMPKα axis. Furthermore, the lactate levels in PANC1 cells co-treated with metformin and FX11 were significantly decreased, while the increased cellular stress led the cells to apoptosis.

**Conclusions:**

Compared with metformin treatment alone, the combination treatment of metformin and FX11 stimulates cellular stress in pancreatic cancer and targets various energy processes that encourage cancer cells to undergo apoptosis. This study provides a novel therapeutic strategy for the treatment of pancreatic cancer.

## Background

Pancreatic cancer is one of the most aggressive and malignant tumors. Most patients with pancreatic cancer are diagnosed at an inoperable stage. Despite improvements in early diagnosis, surgical techniques, and chemotherapy, the 5-year survival rate of patients with pancreatic cancer is approximately 6% [1], and the overall prognosis of patients with pancreatic cancer remains poor; therefore, more effective therapeutic strategies need to be developed [2]. The development of novel target mechanisms is crucial for tumors, like pancreatic cancer, that have become resistant to current chemotherapeutic therapies.

Glucose metabolism, essential for mammalian survival, uses energy in the form of ATP through the oxidation of carbon bonds. The end-product of energy metabolism in mammals may be CO2, which is formed due to the complete oxidation of glucose, or lactate, which is not produced in this way [3]. Unlike healthy cells, cancer cells depend on a process called aerobic glycolysis, known as the Warburg effect. Even in the presence of perfectly functioning mitochondria and sufficient oxygen in the environment, cancer cells rapidly convert glucose into lactate [4]. In the presence of sufficient oxygen and mitochondrial capacity in the cell, pyruvate is normally converted to acetyl-CoA and then enters the Krebs cycle. In the absence of oxygen or excessive glycolysis, pyruvate is reduced to lactate, which is produced by the enzyme lactate dehydrogenase (LDH) [5]. This process recycles NAD+, which is reduced to NADH during glycolysis, thus allowing glycolysis to continue. The conversion of pyruvate to lactate maintains intracellular pH homeostasis and acidifies the extracellular space by removing protons from the glyceraldehyde 3-phosphate dehydrogenase reaction during glycolysis. Studies have shown that lactate in the microenvironment modulates immune cell function and promotes invasion and metastasis, including tumor glycolysis and lactate secretion, in processes that affect cancer-related mortality [6, 7].

Metformin, a widely used antihyperglycemic agent, is also of interest in cancer treatment and prevention. Epidemiologic studies have shown that metformin treatment in type-2 diabetes patients is associated with a reduced risk of several cancers, such as colorectal cancer, prostate cancer, and pancreatic cancer [8, 9]. Based on this evidence, in-vitro and in-vivo studies have demonstrated that the use of metformin as a therapeutic agent can inhibit cell proliferation in cancer cell lines and affect tumor growth in mouse xenografts of cancers, such as prostate cancer, breast cancer and colon cancer [10–12]. Wang et al. demonstrated in their systemic review and meta-analysis that there is also evidence for a significant association between metformin adjuvant treatment and survival benefit for pancreatic cancer patients, suggesting a potentially available option for treatment [9]. Studies on pancreatic cell lines and animals have also shown that metformin inhibits cell proliferation and restricts tumor growth [13–15]. The effect of metformin depends on its ability to alter the energetic state of the cell. The central mechanism of action involves inhibiting respiratory chain complex 1, lowering the energy status of the cell and inhibiting gluconeogenesis. The lower energy status of the cell indicated by the increasing AMP/ATP ratio activates AMP-activated protein kinase (AMPK), which controls significant pathways affecting cell growth. By decreasing protein synthesis and enforcing a metabolic checkpoint during the cell cycle, metformin slows cell proliferation [16, 17]. Studies have shown that cells treated with metformin become energetically inefficient and display increased glycolysis [18]. Molecular mechanisms by which metformin affects tumors: insulin-dependent/independent, ultimately inhibiting the growth of cancer cells. Both of these mechanisms of metformin depend on the activation of AMP-activated protein kinase (AMPK) [19]. AMPK regulates the energy metabolism of cells and is activated by the increase in AMP/ATP. LKB1 is located upstream of AMPK. LKB1 is a serine/threonine kinase previously identified as a tumor suppressor gene [20, 21]. This molecule is the kinase responsible for the phosphorylation of AMPK and is required to activate AMPK in response to energy stress in cell culture [22–24]. As a direct result of AMPK activation, inhibition of the mTOR pathway occurs through phosphorylation and activation of tuberous sclerosis complex 2 (TSC2), a subunit of the TSC1/TSC2 (hamartin/tuberin) complex that negatively regulates mTOR signaling [25, 26]. In many cellular processes involved in energy consumption, mTOR plays a central role in regulating cell growth [27]. Therefore, the inhibition of mTOR through AMPK activation is an important approach for cancer therapy [28].

Aerobic glycolysis, a key feature of metabolic reprogramming in cancer cells, supports rapid and continuous proliferation and growth, maintenance of the cancer stem cell state, survival, and immune system evasion. It plays a role in malignant progression and is also characterized by an increase in key glycolytic enzymes, such as lactate dehydrogenase A (LDHA), which is responsible for converting pyruvate to lactate [29]. Studies have shown that the inhibition of LDHA expression in cancer cell lines results in increased oxygen consumption and reactive oxygen species production, reduced glucose uptake and lactate production, and decreased tumor cell growth [30, 31]. Many human cancer cell lines, such as those from pediatric osteosarcoma, lymphoma, prostate cancer, gallbladder carcinoma, hepatocellular carcinoma, or pancreatic cancer, have been shown to exhibit abnormal overexpression of LDHA, which can also promote cancer proliferation, migration, and invasion [32–35]. Targeting key glycolytic enzymes has also increased interest as a therapeutic intervention option in pancreatic cancer. In-vitro studies have demonstrated that targeting LDHA can reduce the proliferation, migration, and invasion of pancreatic cancer, and in-vivo studies have shown that tumor growth is decreased [32, 36]. In many experiments, FX11 (3-dihydroxy-6-methyl-7-(phenylmethyl)-4-propylnaphthalene-1-carboxylic acid), an NADH competitive inhibitor of LDHA, was used to inhibit the expression of LDHA [34, 36]. Both metformin and FX11 have been studied as therapeutic approaches in cancer treatment, and both have been shown to affect cancer cell energy metabolism [37–39]. However, the effects of their combination on pancreatic cancer cells have not been investigated.

According to the hypothesis of the study, co-administration of FX11 and metformin should target cancer cells through two important mechanisms. First, FX11 decreases the synthesis of ATP through glycolysis, whereas metformin inhibits mitochondrial respiration. An energy crisis may result from this since cells may quickly exhaust their energy stores. Due to their high energy requirements, cancer cells should experience a significant energy shortage that triggers death if both energy generation pathways are simultaneously inhibited [40]. Second, FX11 might lead to the formation of ROS by increasing mitochondrial activity. Particularly in cells that are already under stress, this would set off the apoptotic process. Metformin’s inhibition of mitochondrial respiration might accelerate the process. Based on this assumption, our study combined metformin and FX11 agents to increase the cell death rate in cancer cells, resulting from increased oxidative stress and energy shortage. We evaluated the effects of metformin and FX11, alone or in combination, in a pancreatic cancer cell line and assessed the impact of these agents via several in vitro assays, LDH assays, apoptosis assays, and Western blotting.

## Methods

### Cell Culture and reagents

The human pancreatic cancer cell line PANC-1 was obtained from Yeditepe University Genetic Diagnosis Center (Acibadem, Turkey). The PANC-1 cell line to be used in the study was chosen based on its hypoxic microenvironment, dependency on glycolysis, and easy cultivability. PANC-1 cells were cultivated in DMEM (Dulbecco’s modified Eagle’s medium) supplemented with low glucose, 10% FBS and 1% (w/v) penicillin/streptomycin at 37 °C with 5% CO2. A 0.25% trypsin-EDTA solution was used for subculture of cells. The medium was changed every 2 days, and the cells were passaged when they reached 80% confluence.

Metformin and FX11 with a high purity ratio, were preferred in the present study, and the agents were used according to the manufacturer’s protocol. The dose range and in-vitro administration period used to determine the effect on cell viability were applied based on literature studies; Metformin (Purity: >98%, Fisher Scientific, catalogue number: 2864100) was used in a range of 0.05 mM to 25 mM [41–44], and FX11 (Purity: ≥96%, Calbiochem, catalogue number: 427218) was used in a range of 0.5 µM to 30 µM [45–47].

### Cell viability assay

Cell viability was assessed with the CellTiter 96^®^ Aqueous One Solution Cell Proliferation Assay (Promega, catalogue number: G3581). PANC-1 cells were seeded in 96-well plates and treated with metformin or FX11, and their combinations were investigated to determine their effects on the proliferation and progression of cancer cells. The metformin concentrations used in this study ranged from 0.05 mM to 25 mM, and the FX11 concentrations ranged from 0.5 µM to 30 µM. The MTS assay was performed at 96 h. The absorbance was recorded at 490 nm via a microplate reader.

### Lactate assay

The lactate levels of the experimental groups were measured with a colorimetric lactate assay kit (Cell Biolab Inc., catalogue number: MET-5012). This assay measures lactate in biological samples. Lactate is oxidized by lactate oxidase to pyruvate and hydrogen peroxide. Hydrogen peroxide is then detected with a highly specific colorimetric probe. Horseradish peroxidase catalyzes the reaction between the probe and hydrogen peroxide bound at a ratio of 1:1. The samples and standards were incubated for 30–45 min and then read with a standard 96-well colorimetric plate reader. The samples were compared to a known concentration of lactate standard in a 96-well microtiter plate. To measure the lactate level of the experimental groups, the assay was performed according to the manufacturer’s instructions at the end of 96 h.

### Western blot

Western blotting was performed with AMPKα- and p-AMPKα-specific antibodies via Mini-PROTEAN^®^ Tetra Cell, Mini Trans-Blot^®^ Module, and 22 PowerPac™ Basic Power Supply (Bio-Rad, 1658033) in the laboratory. β-actin was used as an internal control. Total protein isolation from the cell groups was carried out via “RIPA (CST, Cat No: 9806)” supplemented with “phosphatase & protease inhibitor solution (Thermo Scientific, Cat No: 78440)”. The protein concentration was determined via a Pierce BCA Protein Assay Kit (Thermo Scientific, Cat No: 23225). Proteins were separated via 12% SDS‒PAGE (TGX™ FastCast™ Acrylamide Kit, Bio-Rad, Cat no: 1610175) and transferred to a PVDF membrane (polyvinyl fluoride 45 mm, Thermo Scientific, cat no: 88518). The membranes were subsequently blocked in 2.5% bovine serum albumin (BSA; Sigma‒Aldrich, Cat no: A9647) solution. Antigen antibody complexes were formed with a horseradish peroxidase (HRP)-conjugated secondary antibody (Invitrogen Life Technology), and the resulting luminescence was obtained via chemiluminescence irradiation (Clarity Western ECL Substrate Kit, Bio-Rad, Cat no: 1705061). After imaging, the protein bands were analyzed via the “ImageJ (Image Processing and Analysis in Java)” program.

### Apoptosis assay

The apoptosis assay was performed via a PE Annexin V Apoptosis Detection Kit I (BD Pharmingen™) according to the manufacturer’s instructions. PE Annexin V staining precedes the loss of membrane integrity from apoptotic or necrotic processes. Therefore, PE Annexin V staining is used in conjunction with 7-amino-actinomycin (7-AAD) to allow the identification of early apoptotic cells (7-AAD negative, PE Annexin V positive). Living cells with intact membranes exclude 7-AAD, whereas membranes of dead and damaged cells are permeable to 7-AAD. PANC-1 cells were treated with metformin and/or FX11 and DMSO (control) for 96 h, after which the assay was performed according to the manufacturer’s instructions.

### Statistical analysis

The data were statistically analyzed via the ANOVA (unpaired) method. Differences with p values of < 0.05 were considered statistically significant. GraphPad Prism 8 (GraphPad, San Diego, CA, USA) was used for all the statistical analyses. In each experiment, results were obtained by analysis of repeated experiments (*n* = 3).

## Results

### Cell viability assay

**Fig. 1:**
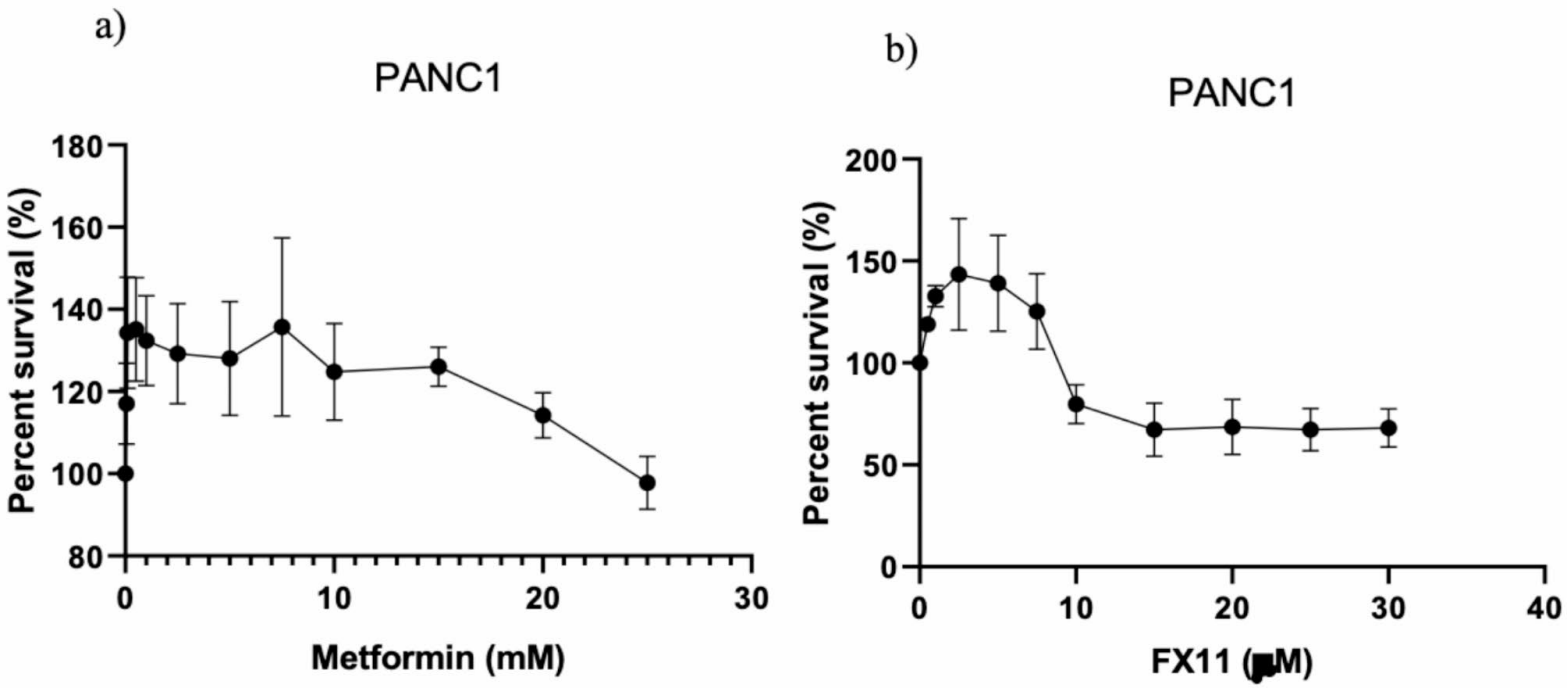
Viability of PANC-1 cells via the MTS assay. **a**) Viability of cells treated with metformin. **b**) Viability of cells treated with FX11

**Fig. 2:**
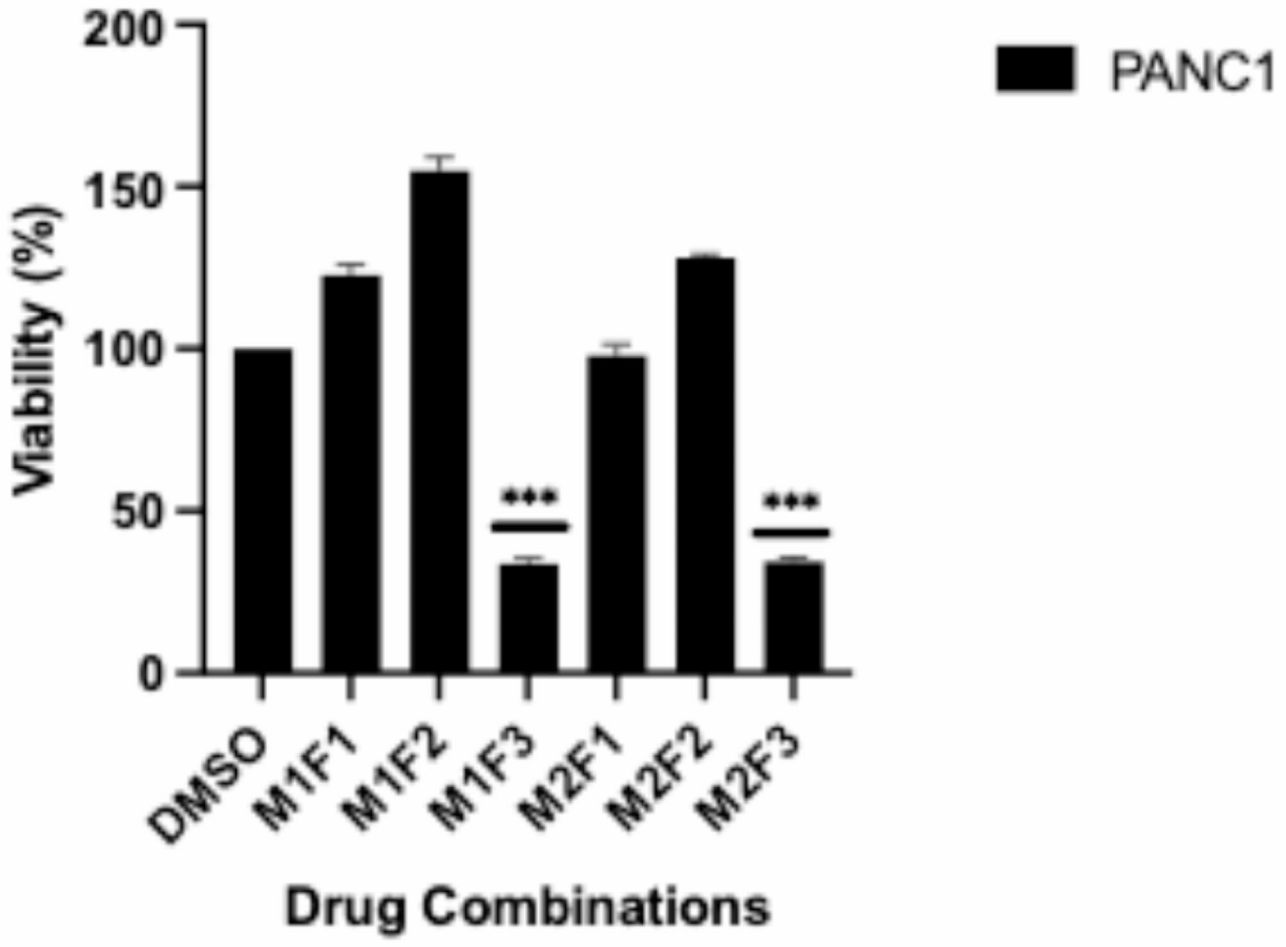
Effects of drug combinations on viability. M: metformin; M1: 0.1 mM (low-dose) metformin; M2: 10 mM (effective-dose) metformin. F: FX11 agents; F1: 2.5 µM (low-dose), F2: 7.5 µM (intermediate-dose), F3: 20 µM (effective-dose). DMSO: Control group. Experiments were performed in triplicate (*n* = 3). Statistical significance: *p* < 0.05

Increasing the metformin dose decreased the viability of PANC-1 cells in an inversely correlated manner (Fig. 1A-B). Similarly, increasing the FX11 dose reduced the viability of PANC-1 cells. Furthermore, various FX11 concentrations were combined with both high and low metformin concentrations. In combination with an effective FX11 dose and two distinct metformin doses, the effects on cell viability were very similar (Fig. 2). The selected doses of metformin or FX11, which affect cell viability when administered alone, were administered in combination. Metformin agent alone has a toxic effect on cell viability when used at high doses (20 mM and above). High doses of FX11 (30 µM and above), on the other hand, did not cause a significant change in cell viability. In addition, the combination of low-dose (0.1 mM) metformin and FX11(20 µM) agents (M1F3 group in Fig. 2) did not have a significant effect on functional experiments (data not shown), although a significant decrease in cell viability occurred even in the presence of lower metformin. Therefore, the condition in which the effective dose of metformin (10 mM) was used both alone and in combination (M2F3 group in Fig. 2) with FX11 was used in further experiments.

### LDH assay

**Fig. 3:**
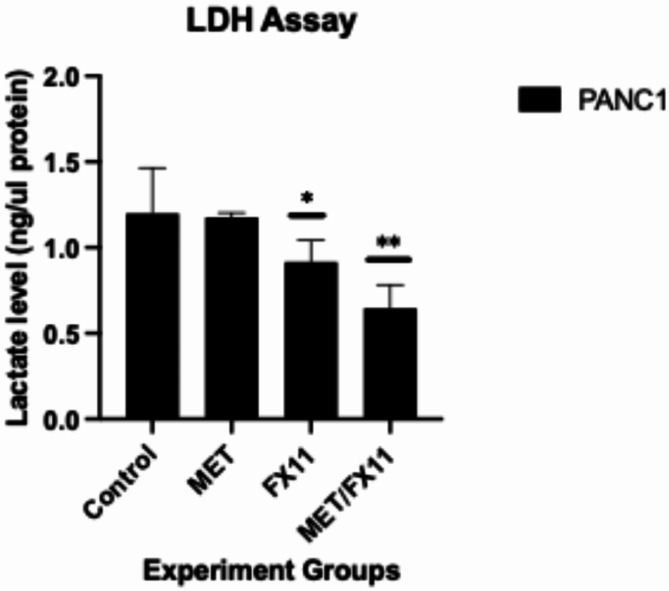
Intracellular lactate levels in PANC-1 cells. MET: metformin; MET/FX11: combination of metformin (10 mM) and FX11 (20 µM). Experiments were performed in triplicate (*n* = 3). Statistical significance: *p* < 0.05

Theoretically, FX11 should reduce lactate production by inhibiting LDH. In combination with metformin, cancer cells produce less pyruvate through lower energy production pathways. Therefore, a significant reduction in lactate levels is expected when these two agents are used together. To test the cellular lactate level, an LDH assay was used in this study. Lactate levels in PANC-1 cells treated for 96 h with metformin (10 mM) and/or FX11 (20 µM) are shown in Fig. 3. Lactate levels were significantly lower than those in the control group (*p < 0.05).

### Western blot

**Fig. 4:**
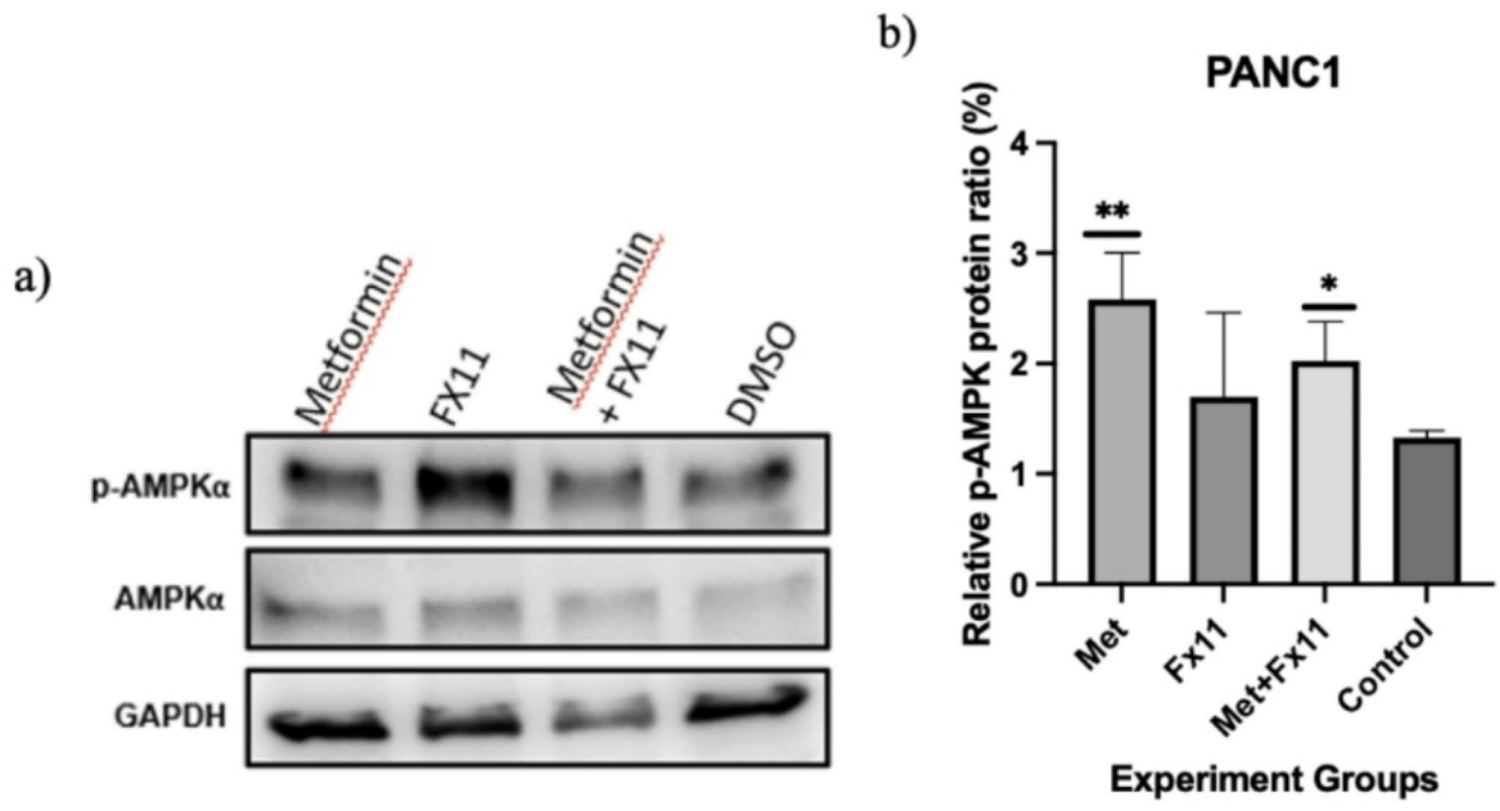
AMPKα protein levels in PANC-1 cells. **a**) Membrane image of the western blot analysis. **b**) Bar charts of the western blot analysis results. Experiments were performed in triplicate (*n* = 3). Statistical significance: *p* < 0.05

The amount of AMPKα protein altered in the experimental groups compared with the control group was determined. β-actin was used as an internal control protein. Compared with that in the control PANC-1 cells, the p-AMPKα protein ratio was greater in the metformin (10 mM) and FX11 (20 µM) combination groups than in the control group (Fig. 4).

### Apoptosis assay

**Fig. 5:**
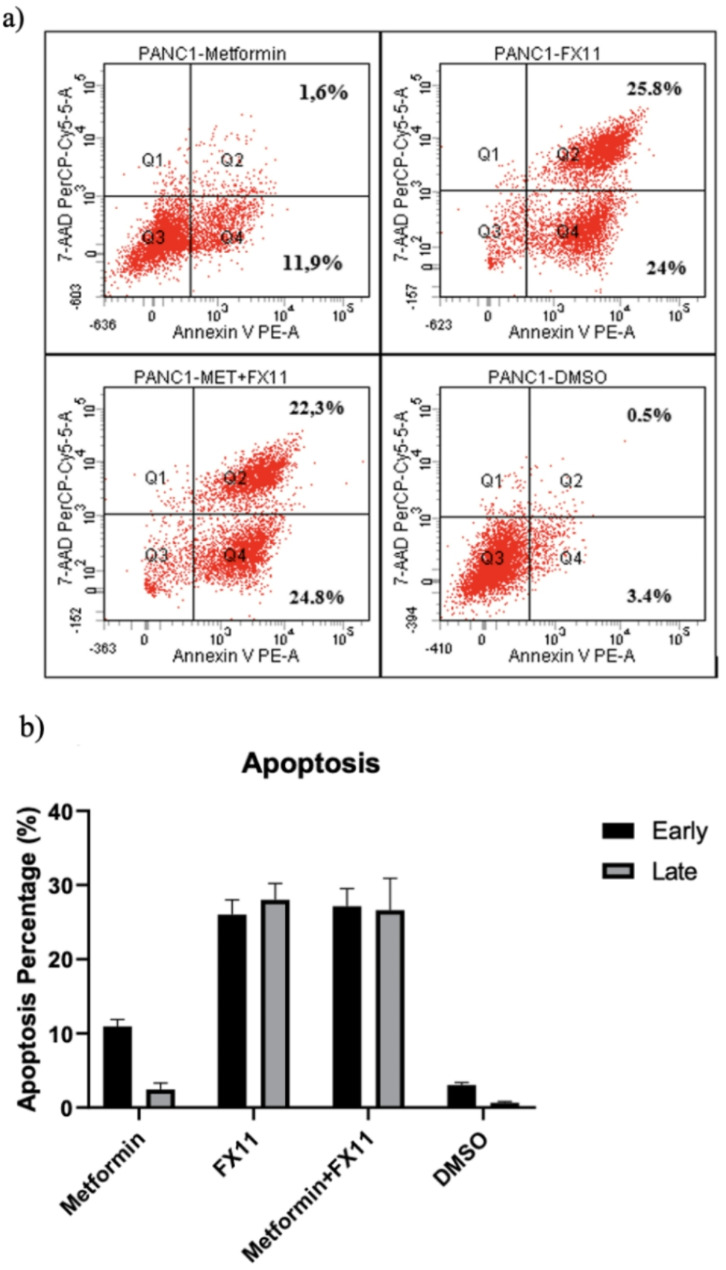
Apoptosis level of PANC-1 cells. DMSO: control group; early: early apoptotic cells; late: late apoptotic cells. **a**) Flow cytometry panel of the apoptosis assay. **b**) Bar charts of the apoptosis assay results. Experiments were performed in triplicate (*n* = 3). Statistical significance: *p* < 0.05

The apoptosis level of cells was investigated via an Annexin V/7-AAD staining assay (Fig. 5). Among the agents applied to the cells for 96 h, metformin (10 mM) induced 11.9% early apoptosis and 1.6% late apoptosis in PANC-1 cells. FX11 (20 µM) induced 24% early apoptosis and 25.8% late apoptosis. The combination of metformin and FX11 induced 24.8% early apoptosis and 22.3% late apoptosis. The results revealed that there were no significant differences between the early and late stages of apoptosis; however, the results revealed that the cells were significantly more likely to undergo apoptosis than the DMSO control group was.

## Discussion

Metformin and FX11 have different effects on cell mechanisms. Elevated cellular glucose metabolism is a parameter that plays an important role in cancer progression. In addition to the antidiabetic properties of metformin, most recent clinical studies have shown that it has a significant effect on cancer progression [48–50]. The main mechanism of action of metformin is to inhibit mitochondrial complex I, which plays a role in the cellular energy supply, making it difficult for the cell to supply energy and inhibiting the mTOR (mammalian target of rapamycin) pathway, which is responsible for growth and proliferation by activating the AMPK signaling pathway. This affects the proliferation of cancer cells by suppressing cell growth and protein synthesis [51, 52]. FX11 is an inhibitor of an enzyme that plays a critical role in enabling cells to obtain energy through glycolysis. Cancer cells frequently utilize the Warburg effect, which enables energy production by converting glucose to lactate and thus can promote rapid energy production. FX11 acts as an inhibitor of the enzyme LDHA, which acts precisely on this mechanism [47, 53]. In the literature, Hua et al. reported that metformin has started to be used in anticancer interventions because of its ‘direct’ effects as an insulin sensitizer as well as its ‘indirect’ effect on cellular energy mechanisms [54]. Another study aimed to increase the anticancer effect of metformin in combination with antitumor agents [55, 56]. In a study investigating the anticancer effects of FX11, the LDHA enzyme was found to be essential for cancer progression. In addition, FX11 treatment reduced tumor volume and slowed progression in lymphoma and pancreatic cancers [57]. In the literature, studies have targeted different energy mechanisms to evaluate their effects on cancer progression. In one of these studies, it was determined that the shift between metabolic enzymes reduced the progression of cancer cells by stressing cancer cells via energy mechanisms [58].

In the experimental stages of our study, we prioritized the choice of cancer type and cell line with appropriate characteristics to evaluate the hypothesis. Pancreatic cancer was therefore one of the most common cancer types with a significant metabolic Warburg effect and glucose dependence [59–62]. In addition, to demonstrate the hypothesis of the study experimentally, the cancer type to be selected had been investigated in terms of microenvironment. Therefore, among the cancer types with elevated LDHA activity and a hypoxic microenvironment, pancreatic cancer was once again prevalent. This investigation was conducted using pancreatic cancer cells based on these selection criteria [63–67]. The PANC1 cell line was used for our study’s pancreatic cancer model due to the based on its therapy resistance character [68–71]. In order to determine the impact of Metformin/FX11 combination therapy in resistant tumors, it was aimed at modeling some of the resistance mechanisms noticed in the clinic using the PANC1 cell line. In addition, PANC-1 cells represent specific biological characteristics of cancer cells, such as glycolysis dependence (Warburg effect). The metabolic pathways of healthy pancreatic cells are very different. Therefore, they might not directly address this study’s concept. Similarly, the combination of FX11 and metformin may be effective in cells with high LDHA activity and glycolysis dependence. Since this mechanism is usually not active in healthy cells, it may not be appropriate to include healthy cells in the study. Therefore, experimental studies were performed with a pancreatic cancer cell line. In further studies, different pancreatic cancer cell lines and cell lines of different cancer types can be included in the study.

In our study, the effects of metformin and FX11 on the viability of pancreatic cancer cells were individually assessed in a dose-dependent manner. However, the combined use of these two agents significantly affected the viability of cancer cells. For this reason, the effects of both alone and in combination were analyzed in all functional experiments. An effective dose of FX11 similarly affects viability at low and high metformin doses. The fact that a lower dose of metformin is required when metformin is used in combination with FX11 suggests that lower doses of metformin could also be investigated in combination. In this study, the same experiments were performed with low-dose metformin (0.01–0.1 mM). However, in these experiments, low-dose metformin did not have an anticancer effect on functional experiments (data not shown).

In recent years, the interest in the anticancer activity of metformin has increased and it has been determined that it induces apoptosis in various types of cancer cells. In these studies, it has been shown that metformin induces apoptosis through critical molecules in cancers such as STAT3, as well as its direct activity on programmed cell pathways [13, 72–76]. The energy mechanism that cancer cells provide for themselves increases their proliferation rate and supports their ability to divide indefinitely [58]. Inhibition of the energy mechanism and increased cellular ROS levels due to inhibition of glycolysis can inhibit the proliferation of cancer cells. This study aimed to target pancreatic cancer cells through several energy mechanisms to restrict their energy consumption and increase their tendency toward apoptosis by increasing cellular stress. For this purpose, cells are targeted with metformin and FX11 to inhibit both the glycolysis pathway and oxidative phosphorylation and to increase the tendency toward apoptosis by increasing ROS production, leading to DNA damage and cellular stress in cells. This strategy is intended to be effective in pancreatic cancer cells that are highly glycolysis dependent and exhibit the Warburg effect. Compared with their counterparts, PANC-1 cells treated with a combination of metformin and FX11 presented significantly lower lactate levels. This decrease in the lactate level in PANC-1 cells suggested that the glycolysis pathway, which is highly dependent on glucose, could be inhibited in these cells. Cell viability was shown to decrease significantly when 20 µM FX11 was administered to PANC-1 cells. An apoptosis assay was performed to determine whether this decrease in the viability of PANC-1 cells was due to a programmed cell death pathway. The results revealed that the cell group that received both FX11 and metformin combination treatment exhibited a significant percentage of apoptotic cells. Early and late apoptotic cells did not differ significantly from one another.

In this study, metformin was used to investigate the mechanism by which it inhibits mitochondrial respiratory chain complex-1. This inhibition alters the energy status of the cell and activates AMP-activated protein kinase (AMPK) [77]. AMPK is a very important sensor for the energy status of the cell. Owing to the increased AMP/ATP ratio in the cell and the imbalance between ATP production and consumption, AMPK is activated. AMPK also controls cell growth in response to changes in energy levels. One of the important pathways regulating growth is the mammalian target of the rapamycin (mTOR) pathway, which is influenced by the LKB1/AMPK axis. The majority of human malignancies have dysregulated mTOR, which is recognized as the primary regulator of nutritional and growth factor inputs in the control of cell division in all eukaryotes. The p-AMPKα/AMPKα protein levels in PANC-1 cells were measured via western blotting because of this crucial axis [39, 78].

The combination of FX11 and metformin in PANC-1 cells resulted in a considerable increase in p-AMPK levels compared with those in control cells. When analyzed along with other experiments, the combined use of FX11 and metformin considerably reduced PANC-1 aggressive behavior, even though the group receiving only metformin presented a greater increase in p-AMPK. These findings indicate that downstream pathways are also affected by metformin and FX11 and that elevated p-AMPK levels could have an effect on the mTOR protein, which is dysregulated in a variety of cancer types.

## Conclusions

In summary, the results obtained in this study, the combination of metformin and FX11 is promising for cancer treatment. Considering that the combination of metformin and FX11 affects the axis that regulates vital functions in the cell, uncontrolled growth and development of cells can be controlled not only in pancreatic cancer but also in many different types of cancer. This mechanism stimulates cellular stress in pancreatic cancer and targets various energy processes that encourage cancer cells to apoptosis. Further studies aim to investigate the in vivo effect of this mechanism.

## Data Availability

All data generated or analyzed during this study are included in this published article.
